# Clinical Implication of Dosimetry of Computed Tomography- and Fluoroscopy-Guided Intrathecal Therapy With Nusinersen in Adult Patients With Spinal Muscular Atrophy

**DOI:** 10.3389/fneur.2019.01166

**Published:** 2019-11-05

**Authors:** Kathrin Kizina, Benjamin Stolte, Andreas Totzeck, Saskia Bolz, Michael Fleischer, Christoph Mönninghoff, Nika Guberina, Denise Oldenburg, Michael Forsting, Christoph Kleinschnitz, Tim Hagenacker

**Affiliations:** ^1^Department of Neurology, University Hospital Essen, Essen, Germany; ^2^Department of Diagnostic and Interventional Radiology and Neuroradiology, University Hospital Essen, Essen, Germany

**Keywords:** lumbar puncture, scoliosis, CT, SMA, radiation exposure

## Abstract

**Background:** Spinal muscular atrophy (SMA) is a genetic disorder that leads to progressive tetraparesis. Nusinersen is the first approved drug for the treatment of SMA and is administered via intrathecal injections. Neuromyopathic scoliosis and spondylodesis can impede lumbar punctures, thus necessitating the use of radiological imaging. Furthermore, dosimetry of this potentially lifelong therapy should be supervised.

**Methods:** Fluoroscopy-assisted or computed tomography (CT)-guided intrathecal injections of nusinersen were performed in adult patients with SMA type 2 and 3. The mean effective dose was compared in patients with and without spondylodesis as well as in those with SMA type 2 and 3. The dosimetry was analyzed in relation to the motor function evaluated with the Revised Upper Limb module (RULM) score and the Hammersmith Functional Motor Scale-Expanded (HFMSE) score.

**Results:** Fifteen patients with SMA type 2 and 3 underwent radiological imaging-assisted intrathecal injections. The mean effective dose per CT-guided injection per patient was 2.59 (±1.67) mSv (*n* = 12). The mean dose area product (DAP) per fluoroscopy-guided injection per patient was 200.48 (±323.67) μGym^2^ (*n* = 3). With increase in the number of injections, the effective dose (*r* = −0.23) (*p* < 0.05) and the DAP (*r* = −0.09) (*p* > 0.05) decreased. The mean effective dose in 4 patients without spinal fusion (SMA type 2) was 1.39 (±0.51) mSv, whereas that in 8 patients with spondylodesis (SMA type 2 and 3) was 3.21 (±1.73) mSv. The mean effective dose in 5 SMA type 2 patients with spondylodesis was 2.68 (±1.47) mSv (*n* = 5) and in 3 SMA type 3 patients was 4.00 (±1.82) mSv. Dosimetry did not show significant correlation with the clinical severity of the disease (RULM score: *r* = −0.045, *p* > 0.05 and HFMSE score: *r* = −0.001, *p* > 0.05).

**Conclusions:** In SMA type 2 and 3 patients undergoing radiological imaging-assisted injections, the effective dose and DAP decreased during therapy with nusinersen. The mean effective dose in patients with spondylodesis was higher than that in patients without spondylodesis. Dosimetry should be monitored carefully in order to detect and prevent unnecessary radiation exposure.

## Introduction

Spinal muscular atrophy (SMA) is an autosomal recessive, inherited, degenerative motor neuron disease with a prevalence of 1 per 10,000 births. Clinically, it leads to progressive muscle atrophy and weakness. Depending on the achieved motor milestones, SMA is distinguished into different phenotypes: infants with SMA type 1 (Werdnig-Hoffmann disease) never develop the ability to sit, and they typically die within the first 2 years because of respiratory failure without ventilatory support (“non-sitters”). Children with SMA type 2 develop the ability to sit unassisted but never learn to ambulate independently (“sitters”). Patients with SMA type 3 (Kugelberg-Welander disease) learn to walk (“walkers”). However, the achieved motor milestones can be lost during the course of the disease. When clinical manifestation begins in adulthood (type 4), patients are typically less affected (1, 2).

In most cases, SMA is caused by a homozygous mutation of the survival of motor neuron 1 gene (SMN1) on chromosome 5q leading to insufficient levels of full-length SMN protein. In the SMN2 gene, a centromeric copy of SMN1, a single C-to-T transition results in production of mostly truncated non-functional SMN protein leading to insufficient development of spinal motor neurons (1, 3–8). As the disease severity is negatively correlated with increase in the number of SMN2 gene copies, SMN2 is the most important disease modifier of SMA (5, 9). Increasing the production of full-length SMN protein is a major target in SMA treatment.

The antisense oligonucleotide (ASO) nusinersen (Spinraza®, Biogen) was approved by the European Medicines Agency (EMA) in May 2017 (10). The approval was based on the pivotal trials “ENDEAR” and “CHERISH” showing that treatment with nusinersen leads to a marked improvement of motor function in SMA type 1 and SMA type 2 patients (2, 11). Nusinersen is capable of increasing the production of full-length SMN protein by modifying the SMN2 gene. Since it is not able to cross the blood-brain barrier, the drug has to be administered via recurrent intrathecal injections (12). The treatment schedule consists of loading doses on day 0, 14, 28, and 64 followed by maintenance doses every 4 months (2, 11). In SMA patients, intrathecal administration of nusinersen can be challenging due to the presence of neuromyopathic scoliosis or spondylodesis. In these patients, CT- or fluoroscopy-guided lumbar punctures may be necessary in order to enable treatment with nusinersen.

As nusinersen is administered over a long term, it is important to give attention to the short- and long-term risks of procedure-related radiation exposure. Herein, we have reported the dosimetric data of CT- and fluoroscopy-guided lumbar punctures associated with the treatment of SMA with nusinersen.

## Methods

### Data Collection

Patients with 5q-associated SMA were treated in accordance with the approval of nusinersen. In the Department of Neurology at the University Hospital Essen (Germany), a total of 70 image-guided injections were performed in 15 SMA patients from August 2017 to August 2018. In detail, 56 CT-guided injections were performed in 12 patients, while 14 injections were performed via fluoroscopy in three patients. By the end of the study, all patients received three injections (*n* = 12), 11 patients received four injections and seven patients received five injections. Two patients received six injections, but because of the small number, we initially excluded them from the results. Patients were aged between 25 and 58 years (average age: 35.1; median: 33.0 years). CT-guided injections were administered to nine patients with SMA type 2 (four without spondylodesis and five with spondylodesis) as well as to three SMA type 3 patients with spondylodesis. Fluoroscopy-guided injections were administered to three patients without spondylodesis. Three board-certified neuroradiologists with work experience of more than 7 years alternately performed the lumbar punctures.

Radiation exposure was monitored by the Radimetrics™ Enterprise Platform software (Bayer Healthcare, Leverkusen, Germany). Based on Monte Carlo simulations techniques, radiation exposure of each image-guided injection (CT and fluoroscopy) was measured, analyzed, and summarized (13). All procedures were performed either on the second-generation dual source CT scanner SOMATOM Definition Flash® or on the third-generation dual source CT scanner SOMATOM FORCE® (both Siemens Healthcare, Forchheim, Germany) with a low radiation dose level (14). Fluoroscopy-guided procedures were performed at the digital flat panel angiography system AXIOM-Artis (Siemens Healthcare, Erlangen, Germany). A measurement of the effective dose according to ICRP 103 (International Commission on Radiological Protection) (15) for CT-guided injections and of the dose area product (DAP) for fluoroscopy-guided injections was performed with regard to the number of injections.

Assuming that radiologically assisted intrathecal injections are more complicated in severely affected patients, radiation exposure was correlated to motor function scores. The Revised Upper Limb Module (RULM) score measures the motor function of the upper extremities. The maximum obtainable score is 37 points (16). The Hammersmith Functional Motor Scale-Expanded (HFMSE) score objectifies the whole-body motor function with a maximum obtainable score of 66 points (17) with higher scores indicating better motor function.

### Statistical Analysis

Statistical analysis was performed with the paired non-parametric *t*-test and unpaired non-parametric test. A *p*-value of <0.05 was considered statistically significant. The correlation coefficient (r) was used to analyze the correlation between changes in the effective dose and the DAP. Linear regression was calculated to compare the effective dose and the DAP regarding the number of injections.

## Results

**Figure 1:**
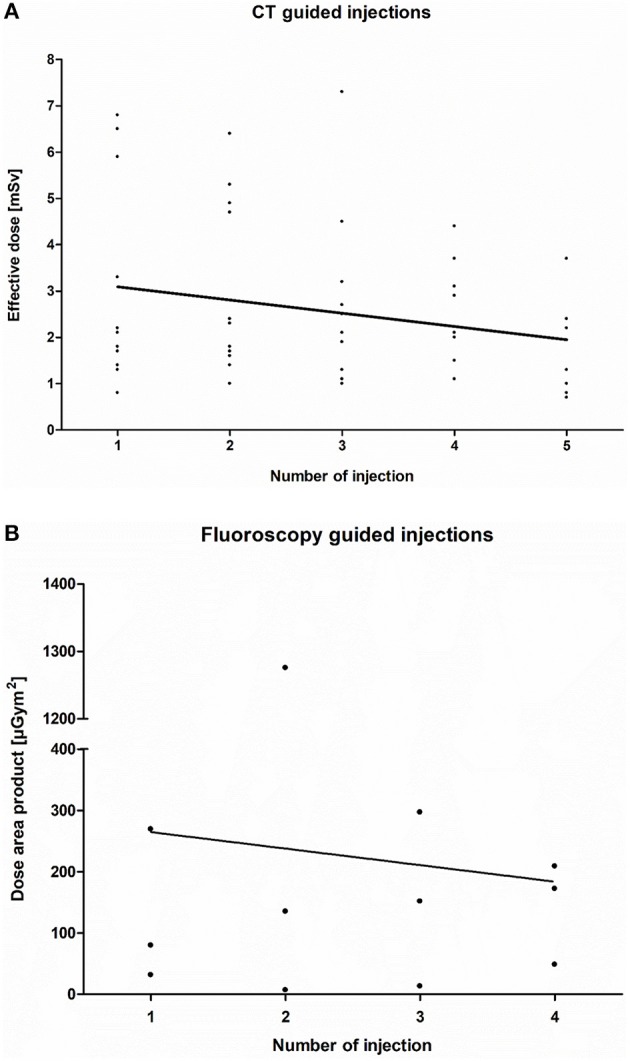
**(A)**Computed tomography (CT)-guided injection: The decrease in the effective dose per patient (*n* = 12) per injection (*r* = −0.22; *p* < 0.05) in the course of Injection 1–5 is shown. **(B)** Fluoroscopy-guided injection: Decrease in the dose area product (DAP) per patient (*n* = 3) per injection (*r* = −0.29; *p* > 0.05) in the course of injection 1–4.

Overall, data from 12 patients with successful CT-guided injections were analyzed. Patient demographics regarding sex, scoliosis, and the presence of spondylodesis as well as the imaging technique used are shown in Table 1. The mean effective dose of each patient per injection was 2.59 (±1.67) mSv (*n* = 12). The median was 2.1 mSv with a maximum of 7.3 mSv and a minimum of 0.7 mSv. The mean effective dose of each injection is presented in Table 2. In the course of the treatment, a significant decrease in the effective dose was observed from the first to the fifth injection with an *r* of −0.23 (*p* < 0.05; Figure 1A).

**Table 1:** Patient demographics.

	**SMA type 2**	**SMA type 3**	**Total**
Number of patients (%)	9 (60%)	6 (40%)	15
Male	3 (33.3%)	6 (100%)	9 (60%)
Female	6 (66.7%)	0 (0%)	6 (40%)
Scoliosis	9 (100%)	6 (100%)	15 (100%)
Spondylodesis	5 (55.6%)	3 (50%)	8 (53.3%)
Fluoroscopy guided	0 (0%)	3 (50%)	3 (20%)
-Success rate^**^–Fluoroscopy guided	–	3^*^	3^*^
Computed tomography (CT) guided	9	3	12
-Success rate^**^–CT guided	9^**^	3	12

* *In one patient, a failed fluoroscopy was switched to a successful CT-guided lumbar puncture*.

** *Defined as successful injection per procedure. In two additional patients, CT-guided injection was not successful*.

**Table 2:** Mean effective dose related to the number of CT-guided injections.

**Number of injections**	**Mean effective dose [mSv]**	**Number of patients (*n*)**
1	2.93 (± 2.19)	12
2	2.94 (± 1.84)	12
3	2.61 (± 1.81)	12
4	2.31 (± 1.08)	11
5	1.73 (± 1.10)	7

In three SMA type 3 patients with only mild scoliosis, nusinersen was injected via fluoroscopy (Figure 1B). The mean DAP of each patient per injection was 200.48 (±323.67) μGym^2^, with a median of 121.80 μGym^2^, a maximum of 1275.70 μGym^2^, and a minimum of 6.86.00 μGym^2^. The linear regression during the course of these injections showed an r of −0.09 with no significant correlation between the DAP and the number of injections (*p* > 0.05). The mean DAP for each injection is shown in Table 3.

**Table 3:** Mean dose area product related to the number of fluoroscopy-guided injections.

**Number of injections**	**Mean dose area product [μGym^**2**^]**	**Number of patients (*n*)**
1	126.94 (±125.67)	3
2	472.6 (±698.46)	3
3	153.89 (±141.80)	3
4	143.38 (±84.04)	3

**Figure 2:**
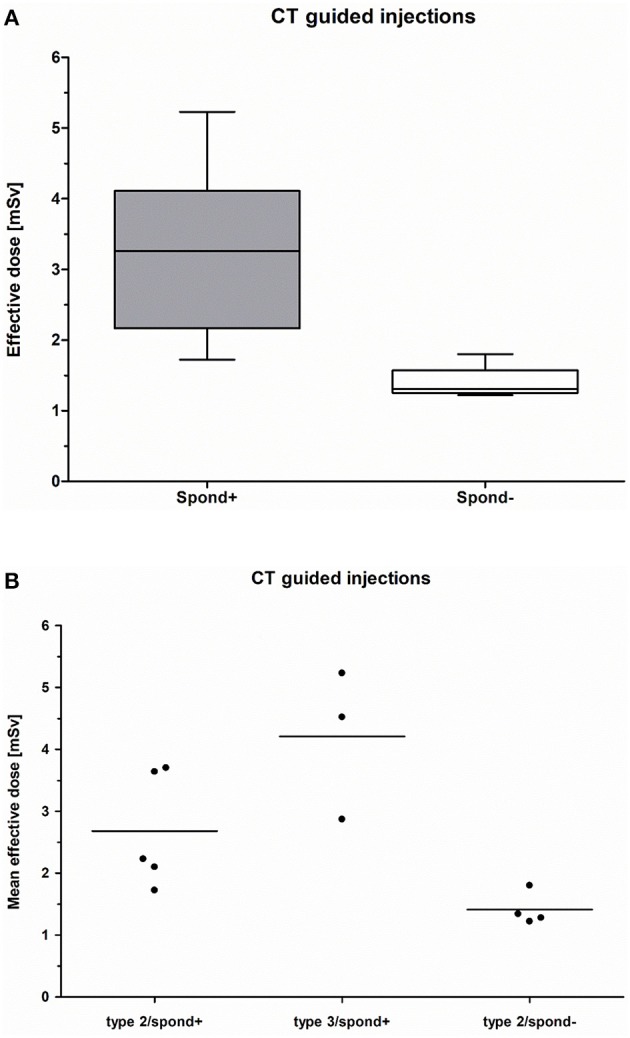
**(A)** CT-guided injection: Mean effective dose of patients with spondylodesis (spond+; *n* = 8) compared to those without spondylodesis (spond–; *n* = 4). **(B)** CT-guided injection: Comparison of the mean effective dose of patients with spondylodesis and SMA type 2 (spond+; *n* = 5), patients with spondylodesis and SMA type 3 (spond+; *n* = 3), and patients without spondylodesis and SMA type 2 (spond–; *n* = 4).

Comparing the effective dose of CT-guided lumbar punctures between patients with and without spondylodesis, the mean effective dose in patients without spinal fusion for each injection (SMA type 2) was 1.39 (± 0.51) mSv (*n* = 4). The median was 1.4 mSv, with a maximum of 2.4 mSv and a minimum of 0.7 mSv, while patients with spondylodesis (SMA type 2 and 3) (*n* = 8) reached an average of 3.21 mSv (±1.73 mSv) with a median of 2.7 mSv, a maximum of 7.3 mSv, and a minimum of 1.1 mSv (Figure 2A). By subclassifying all patients with spondylodesis into SMA type 2 and SMA type 3 groups, the mean effective dose of each patient per CT-guided injection was found to be 2.68 (±1.47) mSv (*n* = 5) with a median of 2.15 mSv, a maximum of 6.8 mSv, and a minimum of 1.1 mSv in SMA type 2, whereas the mean effective dose in patients with SMA type 3 was 4.00 (±1.82) mSv (*n* = 3) per injection with a median of 3.7 mSv, a maximum of 7.3 mSv, and a minimum of 1.3 mSv (Figure 2B).

**Figure 3:**
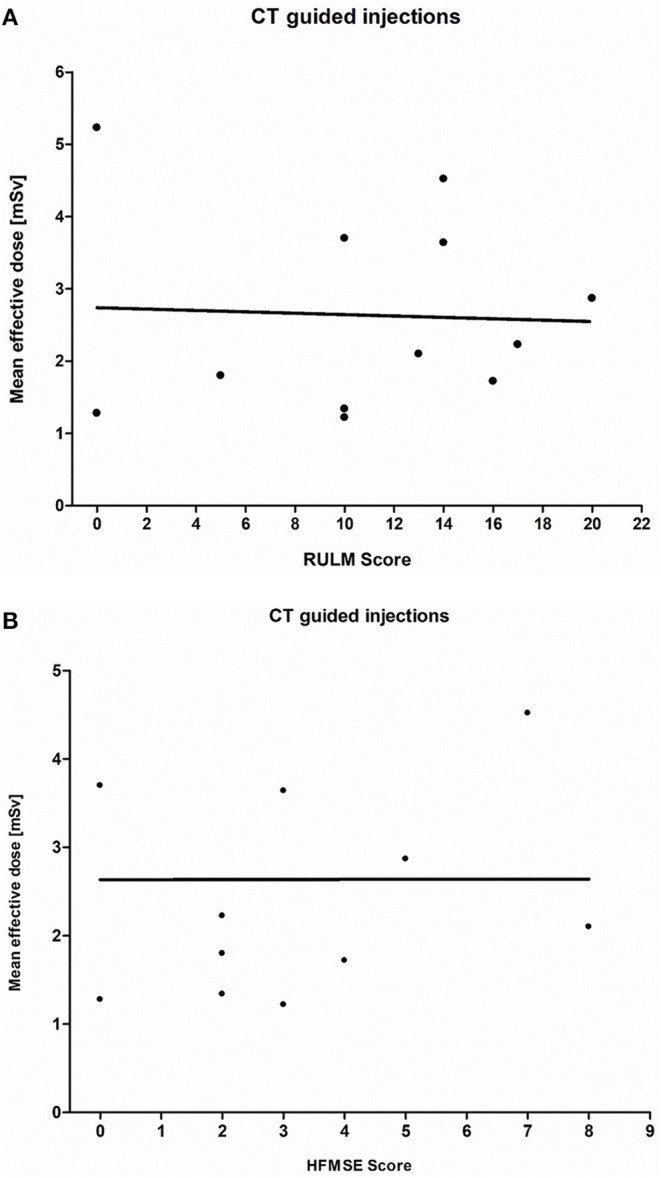
**(A)** CT-guided injection: Presentation of the RULM score (SMA types 2 and 3; *n* = 12; *r* = −0.045; *p* > 0.05). A wide scattering and no correlation between the mean effective dose and RULM score are shown. **(B)** CT-guided injection: Presentation of the HFMSE Score (SMA type 2 and 3; *n* = 12; *r* = −0.001; *p* > 0.05). A wide scattering and no correlation between the mean effective dose and HFMSE Score are shown.

The average RULM score in all SMA patients (*n* = 15) was 12 (±7) with a median of 13, a maximum of 22, and a minimum of 0 points. The mean RULM score in 12 patients with CT-guided injections was 11 (±6) with a median of 12, a maximum of 20, and a minimum of 0 points (Figure 3A). The absolute data are shown in Table 4. With respect to the RULM score in patients with CT-guided injections, *r* was −0.045 with no significant correlation between radiation exposure and RULM score (*p* > 0.05). Three patients who underwent fluoroscopy guided injections had a mean RULM score of 18 (±5) with a median of 20, a maximum of 22, and a minimum of 12 points without any correlation between disease severity and radiation exposure because of the small number of patients in the sample (*n* = 3).

**Table 4:** Correlation of clinical scores and mean effective dose per patient.

**No. of patient**	**1**	**2**	**3**	**4**	**5**	**6**	**7**	**8**	**9**	**10**	**11**	**12**
Mean effective dose [msv]	4.52	2.87	1.72	1.28	1.22	1.34	3.64	3.7	2.1	1.8	5.23	2.23
RULM Score	14	20	16	0	10	10	14	10	13	5	0	17
HFMSE Score	7	5	4	0	3	2	3	0	8	2	0	2

The average HFMSE score in all SMA patients (*n* = 15) was 5 (±4) with a median of 3, a maximum of 15, and a minimum of 0 points. The mean HFMSE score in 12 patients with CT-guided injections was 3 (±3) with a median of 3, a maximum of 8, and a minimum of 0 points (Figure 3B). The absolute data are shown in Table 4. Regarding the HFMSE score in patients with CT-guided injections, *r* was −0.001 with no significant correlation between radiation exposure and HFMSE score (*p* > 0.05). Three patients who received fluoroscopy-guided injections had a mean HFMSE score of 11 (±4) with a median of 12, a maximum of 15, and a minimum of 7 points without a correlation between disease severity and radiation exposure because of the small number of patients in the sample (*n* = 3).

## Discussion

Image-guided lumbar punctures enable treatment with nusinersen for adult SMA type 2 and type 3 patients with severe disease-related anatomical spine alterations (18). During the course of the treatment, there is a significant decrease in the mean effective radiation dose for CT-guided injections. Fluoroscopy-guided treatment shows similar results for the DAP, which is limited by the small number of patients with no significant correlation between the DAP and the number of injections. We selected CT- and fluoroscopy-guided injections based on the degree of the disability, especially the extent of the scoliosis. All patients with spondylodesis received a CT-guided injection. Patients whose degree of disability was too low for a CT-guided injection but too high for a lumbar puncture without radiological support received a fluoroscopy-guided injection (18). Reduction in radiation exposure during the treatment may be based on procedural aspects. With technical information from a prior successful lumbar puncture (i.e., needle position, lumbar level, and angle of puncture) the number of treatment procedures may increase over time due to less radiation exposure from puncture failures. Importantly, in this 1-year study, we were only able to point out a relatively small number of patients. Nevertheless, these patients reflect a real-world perspective of treatment with nusinersen.

Regarding the effective dose for each patient, we observed a large scattering between individual injections. The following three aspects have to be discussed. First, during the course of treatment, the patients did not have the same position in the gantry for each injection, which makes the successful administration of nusinersen technically challenging. Due to extreme scoliosis, the patient's position is sometimes more lateral than prone, which may have influenced the accessibility. Metal artifacts caused by dorsal stabilization may complicate finding the right needle position. Second, saved images and data from successful previous injections show the position and angle of the needles, which is advantageous; this is timesaving and provides a learning opportunity for the interventionalist. Nevertheless, there is no assurance that the needle puncture, performed by the neuroradiologist, is in the exact same position. Third, individual skills of performing neuroradiologists (*n* = 4) may interfere with the radiation exposure.

The mean effective radiation dose for patients with spondylodesis, regardless of the SMA type, was higher than that for patients without a spinal fusion (Figure 2A), which may have been mainly due to the automatic dose upregulation attributable to the higher noise and beam hardening artifacts of spinal fusion devices (19, 20). Another reason for the higher dose might be the more complicated CT-guided administration of nusinersen in patients with severe scoliosis and spinal fusion.

Patients with SMA type 2 are clinically more disabled than patients with SMA type 3 (2, 5, 18). Therefore, we assumed that the effective dose in patients with SMA type 2 and spondylodesis might be higher due to more complicated injection procedures. With limited evidence due to the small number of SMA 3 patients with spondylodesis, our data of patients with SMA type 2 and spondylodesis show a lower effective dose for CT-guided injections compared to patients with SMA type 3 and spondylodesis (Figure 2B). A comparison of the mean effective dose between these two groups is not appropriate due to the small number of patients with SMA type 3 and spondylodesis.

All the patients in our cohort who received CT-guided nusinersen therapy were wheelchair-dependent; the HFMSE score (17). which evaluates whole-body motor function (Figure 3B), may be an insufficient parameter, and the RULM score (16) which evaluates upper limb function in more detail, may be more useful than HFMSE. The clinical deviation in our cohort of patients with SMA was more significant on the RULM than with the HFMSE. Patients with a more severe clinical disability, and therefore lower RULM and HFMSE scores, did not show a lower effective dose for CT-guided injections (Figures 3A,B). Hence, the effective dose may not be affected by the type of SMA and the clinical grade of disability.

Nusinersen therapy is long-term, with a maintenance dose of 3 injections per year; therefore, the radiation exposure should be precisely supervised (21–23). Two possible types of radiation side-effects have to be considered; stochastic radiation effects, which are characterized by incidental damage (e.g., cancer and genotoxic effects) and might appear with a risk of 4.1%/Sv. These effects are independent of the effective dose. In contrast, deterministic radiation effects are characterized by the threshold doses, and particularly affect the reproductive organs, with possible manifestations such as erythema or tissue necrosis (15, 24–27). Exposure to doses >500 mSv may induce the manifestation of the clinical symptoms of radiation exposure, mainly in the hematopoietic or lymphatic system. At 1,000 mSv, radiation-induced skin changes are noted. At >3,000 mSv, the mitotic rate is irreversibly arrested (28, 29). The average dose of radiation exposure for people living in Germany was 3.9 mSv in 2009 (15). In comparison, lumbar spine radiography has an effective dose of 0.6–1.1 mSv, whereas a lumbar spine CT scan has an effective dose of 4.8–8.7 mSv (11). Relatively, the effective doses for CT-guided lumbar punctures in our study were not higher than those for patients with a correct anatomical spine and intrathecal treatment (22, 30).

To date, no injectable therapeutic regimen involves repetitive radiation exposure over time. Considering the long-term exposure, periodic prophylactic mammography screening of women at a higher risk of breast cancer might be, with regard to dosimetry, to a limited extent comparable with long-term nusinersen treatment. Bilateral mammography in two imaging planes has a mean effective dose of 0.2–0.4 mSv, but it has to be considered that both tissues (breast and spine) have distinct radiation sensitivities (24, 31–33). Radiation-induced breast cancer risk decreases with the age at exposure; therefore, for young woman, periodic, long-term screening seems less favorable (33, 34). In comparison, radiology-assisted injections of nusinersen in younger patients have a higher lifetime accumulation of radiation exposure. A second example is the recurrent lumbar punctures for intrathecal chemotherapy in patients with cancer. In this case, the advantages of cancer therapy outweigh the disadvantage of radiation exposure. Therefore, periodic low radiation exposure is generally accepted (30). Another example include patients experiencing back pain who are treated with recurrent CT-guided lumbar nerve root injections with significant benefit (35–37).

A point to consider is the young age of our patients with adult SMA. In general, the risk of radiation-induced cancer depends, among other things, on sage and sex, as well as on the exposure to other carcinogens and promotors that may interact with radiation. Therefore, generally, some patients may or may not develop cancer despite exposure to similar dose of radiation. The younger the patients, the longer the radiation exposure with regard to long-term nusinersen therapy, which results in higher lifetime accumulation of radiation exposure (15, 29, 38, 39). Because the path of the radiation beam is incident at the same area each time, organs in the lower abdomen are the most affected (14). Therefore, the effective dose and DAP should be sufficient yet low as possible for all patients.

In two patients with SMA patients, CT-guided injection of nusinersen was not possible because of spondylodesis and a history of multiple spinal operations with ossification of the puncture site. These two patients were excluded from this study because of unsuccessful lumbar puncture. Because of these complications and the periodic radiation exposure, comparable alternatives to radiology-guided lumbar puncture should be discussed extensively. For patients with unsuccessful CT-guided lumbar puncture, off-label use of an intrathecal catheter system might be considered (40). Another possible intervention is the use of an Ommaya reservoir (41, 42). Till date, it remains unclear whether the efficacy of nusinersen is similar to that of intrathecal injections (43). Therefore, this might be a future alternative for patients with severely altered spinal structure anatomy, without any indication for successful CT-guided therapy, to get nusinersen therapy. A radiation-free and promising method could be MRI-guided therapy with a special equipment (44–46). It is an advantageous alternative especially in young patients who wish have children in future and directs less radiation exposure to the reproductive organs (29, 34, 47). Important disadvantages in this study are the severe technical artifacts caused by our patient's spinal fusion. Another technical modality for radiation-free injection of nusinersen is ultrasound-guided cervical puncture (48); however, cervical injection of nusinersen is considered an off-label use because the EMA only approves lumbar intrathecal injection (10).

No orally administered drugs have been approved for the treatment of SMA; however, the use of risdiplam (RG7916) is being tested in a clinical trial (49). Moreover, oral drug therapy is a radiation-free method without the need for hospitalization, and therefore, confers a gain in the quality of life. Furthermore, there is a major focus on gene therapy, such as onasemnogen abeparvovec, a viral vector with DNA encoding for the survival of motor neuron (SMN) protein, which has been approved by the FDA for treatment of SMA type 1 (50).

## Conclusions

We demonstrated the dosimetry of CT-guided and fluoroscopy-assisted injections of nusinersen over a 1-year period in adult SMA patients. For most patients with severe scoliosis and spondylodesis, these procedures are the only feasible treatment options with nusinersen (10). Overall, our results reveal a decrease in the mean effective dose and the DAP related to the course of injections. The effective dose per injection for patients with scoliosis and spinal fusion was higher than that for patients without spondylodesis. Patients with SMA type 2 have a more severe clinical constraint than those with SMA type 3. Nevertheless, patients with SMA type 2 and spondylodesis compared to those with SMA type 3 and spondylodesis received a lower effective dose per injection with no correlation between the clinical severity and effective radiation dose.

However, radiology-guided lumbar puncture may be the only feasible technical application form for nusinersen in SMA patients with severe scoliosis. The radiation exposure for this long-term therapy must be precisely supervised, while radiation-free or reduced treatment options should be further evaluated.

## Data Availability Statement

All datasets generated for this study are included in the article/supplementary material.

## Ethics Statement

This study was approved by the Ethics Committee at the University Duisburg-Essen, Germany (approval number: 18-8071-BO). Written informed consent was obtained from each patient prior to study inclusion.

## Author Contributions

KK drafted the manuscript and analyzed the clinical data. KK, BS, SB, AT, and TH were responsible for patient recruitment, clinical examination, and nusinersen treatment. MFl performed statistical analysis of the data. CM performed CT- or fluoroscopy-guided injections and performed data analysis. DO and NG analyzed dosimetry data. MFo and CK provided substantial input for data interpretation and manuscript. TH is responsible for the study development and supervision for data analysis and manuscript drafting.

### Conflict of Interest

CK and TH received travel reimbursement and speaker honoraria and declare advisory board attendance from Biogen. The remaining authors declare that the research was conducted in the absence of any commercial or financial relationships that could be construed as a potential conflict of interest.

## Abbreviations

ASO: Antisense oligonucleotide
CT: Computed tomography
DAP: Dose area product
HFMSE: Hammersmith Functional Motor Scale-Expanded
ICRP: International Commission on Radiological Protection
mSv: Millisievert
RULM: Revised Upper Limb Module
SMA: Spinal muscular atrophy
SMN: Survival of motor neuron.
